# Age differences in the intrinsic functional connectivity of default network subsystems

**DOI:** 10.3389/fnagi.2013.00073

**Published:** 2013-11-14

**Authors:** Karen L. Campbell, Omer Grigg, Cristina Saverino, Nathan Churchill, Cheryl L. Grady

**Affiliations:** ^1^Rotman Research Institute, BaycrestToronto, ON, Canada; ^2^Department of Psychology, University of TorontoToronto, ON, Canada; ^3^Department of Medical Biophysics, University of TorontoToronto, ON, Canada; ^4^Department of Psychiatry, University of TorontoToronto, ON, Canada

**Keywords:** fMRI, default network, subsystems, resting state, functional connectivity, aging

## Abstract

Recent work suggests that the default mode network (DMN) includes two core regions, the ventromedial prefrontal cortex and posterior cingulate cortex (PCC), and several unique subsystems that are functionally distinct. These include a medial temporal lobe (MTL) subsystem, active during remembering and future projection, and a dorsomedial prefrontal cortex (dmPFC) subsystem, active during self-reference. The PCC has been further subdivided into ventral (vPCC) and dorsal (dPCC) regions that are more strongly connected with the DMN and cognitive control networks, respectively. The goal of this study was to examine age differences in resting state functional connectivity within these subsystems. After applying a rigorous procedure to reduce the effects of head motion, we used a multivariate technique to identify both common and unique patterns of functional connectivity in the MTL vs. the dmPFC, and in vPCC vs. dPCC. All four areas had robust functional connectivity with other DMN regions, and each also showed distinct connectivity patterns in both age groups. Young and older adults had equivalent functional connectivity in the MTL subsystem. Older adults showed weaker connectivity in the vPCC and dmPFC subsystems, particularly with other DMN areas, but stronger connectivity than younger adults in the dPCC subsystem, which included areas involved in cognitive control. Our data provide evidence for distinct subsystems involving DMN nodes, which are maintained with age. Nevertheless, there are age differences in the strength of functional connectivity within these subsystems, supporting prior evidence that DMN connectivity is particularly vulnerable to age, whereas connectivity involving cognitive control regions is relatively maintained. These results suggest an age difference in the integrated activity among brain networks that can have implications for cognition in older adults.

## INTRODUCTION

Over the past decade, there has been growing interest in the default mode network (DMN), both as a set of regions active during spontaneous thought and less active during externally driven tasks (Shulman et al., 1997; Gusnard et al., 2001; Raichle et al., 2001), and as a functionally connected set of regions at rest and during task performance (Greicius et al., 2003; Fox et al., 2005; Buckner et al., 2008; Grigg and Grady, 2010a; Spreng and Grady, 2010; Allen et al., 2011). Although the precise function of the DMN is still debated, it has been implicated across a wide variety of tasks that require internally directed thought, such as autobiographical memory (e.g., Svoboda et al., 2006; Spreng et al., 2009), future projection (e.g., Addis et al., 2007), mind wandering (e.g., Christoff et al., 2009; Smallwood et al., 2013), and social cognition more generally (e.g., Grigg and Grady, 2010a; Mar, 2011; Kubit and Jack, 2013). One proposal that brings many of these aspects together (Buckner and Carroll, 2006) is that the DMN is involved in projecting the self through time (forward and backward), space (e.g., navigation) and into social situations involving others (theory of mind). Although this has appeal in attempting to explain the wide variety of processes that implicate the DMN, the central process mediated by the DMN remains elusive. Nevertheless, given that it is altered in a variety of disorders [e.g., Alzheimer’s disease (AD), autism, depression], is related to structural connections in the brain, and can be examined at rest without requiring task compliance, the DMN has become an attractive target for many research groups.

Recently, there have been suggestions that the DMN consists of a core set of two areas, the ventromedial prefrontal cortex (vmPFC) and the posterior cingulate cortex (PCC, Fox et al., 2005; Buckner et al., 2008; Toro et al., 2008), and at least two subsystems that are brought online when needed. These are the medial temporal lobe (MTL) subsystem, which is thought to underlie memory reconstruction and future projection (Schacter et al., 2007; Buckner et al., 2008), and the dorsomedial PFC (dmPFC) subsystem, which is thought to mediate self-referential thought more generally (Andrews-Hanna et al., 2010; Andrews-Hanna, 2012).

Support for this idea was reported in a recent study by Andrews-Hanna et al. (2010) that examined the correlations between eleven predefined regions and used graph analysis and hierarchical clustering to parse those regions into subsystems based on relative strength of the correlations. This analysis showed that both the MTL and dmPFC were strongly connected to the core nodes of the DMN, but also interacted with distinct sets of regions (subsystems) that were not inter-related. These subsystems included other DMN regions, as well as areas outside the DMN. This was especially true for the dmPFC subsystem, which included numerous prefrontal regions involved in cognitive control (e.g., Petrides et al., 1993; Badre and D’Esposito, 2007; Vincent et al., 2008; Spreng et al., 2013). Furthermore, an experiment designed to highlight these subsystems showed functional distinctions between them (Andrews-Hanna et al., 2010). The dmPFC subsystem was selectively activated when thinking about oneself in the present and the MTL subsystem was selectively activated when thinking about oneself in the past or projected into the future.

In addition to this evidence for MTL and dmPFC subsystems, another recent study by Leech et al. (2011) showed that different parts of the PCC might participate in distinct sub-networks. In the Leech study, the PCC was subdivided into ventral (vPCC) and dorsal (dPCC) regions that were functionally connected with DMN regions and dorsal attention regions, respectively, depending on the level of task demand. The authors suggested that the vPCC is directly involved in default mode cognitive processing, as it shows strong connectivity with the DMN at rest, but reduced integration as task difficulty increases. The dPCC, on the other hand, is proposed to act as a switch between the DMN and cognitive control regions (Cole and Schneider, 2007), showing the opposite pattern of greater integration with the DMN and decreased integration with cognitive control areas as task difficulty increases. The authors proposed a model of two functional subdivisions of the PCC, such that dPCC should be more strongly functionally connected with control regions, whereas the vPCC should be more strongly connected to other DMN regions, such as the MTL.

The aim of this study was to examine age differences in resting state functional connectivity in these DMN subsystems. Previous work has shown consistently that relative to younger adults, older adults show reduced functional connectivity within the DMN at rest (Andrews-Hanna et al., 2007; Damoiseaux et al., 2008; Esposito et al., 2008; Grady et al., 2010), as well as less pronounced deactivations during cognitive tasks (Lustig et al., 2003; Grady et al., 2006; Persson et al., 2007; Miller et al., 2008; Park et al., 2010). In addition, several studies have identified anterior and posterior components of the DMN and shown age reductions in functional connectivity for both (Damoiseaux et al., 2008; Sambataro et al., 2010). There have been fewer studies looking at age differences in large-scale networks related to cognitive control, such as the frontoparietal control network (Vincent et al., 2008; Spreng et al., 2013) or the salience network (Seeley et al., 2007), and the results of these studies are mixed. Some studies have reported rather widespread age-related reductions of functional connectivity in control networks (Allen et al., 2011; Thomas et al., 2013), whereas others have found age reductions only in some regions (Voss et al., 2010; Campbell et al., 2012a; Onoda et al., 2012), or even increased functional connectivity between some control regions in older relative to young adults (Grady et al., 2010; Rieckmann et al., 2011; Tomasi and Volkow, 2012). Relative sparing of functional connectivity in areas important for cognitive control is consistent with evidence that older adults often engage control regions, such as prefrontal and parietal cortex, to a greater extent than do younger adults during a variety of cognitive tasks (e.g., Rajah and D’Esposito, 2005; Reuter-Lorenz and Cappell, 2008; Grady, 2012).

To date, however, the influence of age on the different DMN subsystems described here has not been examined. Given the evidence of differential involvement of the subsystems in cognitive processes involving the self and in sensitivity to task difficulty, both of which are influenced by aging (Gutchess et al., 2007, 2010; Persson et al., 2007; Grady et al., 2012), it is important to explore these subsystems in older adults. Thus, we contrasted the resting state data from older and younger adults to determine whether there are age differences in the common functional connectivity of the MTL/dmPFC and vPCC/dPCC, involving the DMN as a whole, and in the subsystems uniquely connected to the four areas of interest. To the extent that the subsystems involve DMN areas we expected to see weaker functional connectivity in older adults; subsystems recruiting control-related areas were expected to show maintained functional connectivity with age.

To directly compare the whole-brain patterns of connectivity involving these four regions (MTL, dmPFC, vPCC, and dPCC), and confirm that there are both common and unique aspects of functional connectivity, we used partial least squares (PLS) and a seed-based approach to assess in a multivariate framework the functional connectivity of these regions. PLS (Krishnan et al., 2011) allows for the direct and simultaneous assessment of similarities and differences in whole-brain patterns of functional connectivity for multiple seeds and is ideally suited to address this type of question. We carried out two analyses, one to identify common and unique patterns of functional connectivity for the MTL and dmPFC and one to assess these patterns for the vPCC and dPCC. Because we were interested in examining these subsystems as they are currently defined in the literature, we used as seeds the regions published by Andrews-Hanna et al. (2010), and those reported by Leech et al. (2011). We also assessed the similarity between the patterns of functional connectivity that were obtained in the two analyses.

## MATERIALS AND METHODS

### PARTICIPANTS

Participants were 45 younger (18–29 years; *M* = 22.4, SD = 3.1; 23 males) and 39 older adults (60–83 years; *M* = 69.0, SD = 5.2; 15 males). None of the participants had any history of psychiatric or neurological disorder, drug or alcohol abuse, or any systemic disease that might compromise cognitive function or blood flow (e.g., diabetes, untreated hypertension, cardiovascular disease). All participants were right handed, had normal or corrected to normal vision, and older adults scored in the normal range on the Mini Mental Status Exam (>26) (Folstein et al., 1975). The older adults were more educated than the younger adults [old *M* = 16.0, SD = 2.5; young *M* = 14.6, SD = 1.9; *t*(82) = 2.8, *p* <0.01]. Prior to participation, written informed consent was obtained from all participants. The consent form and study were approved by the Research Ethics Board of Baycrest Centre.

### IMAGE ACQUISITION AND PREPROCESSING

Participants were scanned using a Siemens Trio 3-T scanner. Anatomical scans were acquired with a 3D MP-RAGE sequence (TR = 2s, TE = 2.63 ms, FOV = 25.6 cm^2^, 256 × 256 matrix, 160 slices of 1 mm thickness). Each participant performed a 5 min resting state scan (eyes closed) as part of a longer experimental procedure, which included other functional runs. The participants came from three separate experiments, two of which had the resting state run as the first run of the session (14 young and 15 old from unpublished data; 19 young and 15 old from Grigg and Grady, 2010a), and one in which the resting run was obtained after two runs of cognitive tasks (12 young and 9 old from Campbell et al., 2012a)^1^. The resting state run was acquired with an EPI sequence (170 volumes, TR = 2s, TE = 30 ms, flip angle = 70°, FOV = 20 cm^2^, 64 × 64 matrix, 30 slices of 5 mm thickness, no gap). Measures of pulse and respiration were obtained during the scan.

Preprocessing of the image data was performed with Analysis of Functional Neuroimages (AFNI, Cox, 1996). This included physiological motion correction, rigid motion correction, spatial normalization to Montreal Neurological Institute (MNI) space, and smoothing with an 8 mm Gaussian filter (the final voxel size was 4 mm × 4 mm × 4mm). We also regressed out the white matter and CSF time series from each voxel time series (Grady et al., 2010).

Because motion has recently been shown to influence measures of functional connectivity (Power et al., 2012; Van Dijk et al., 2012), we took an additional step and removed images that appeared to be influenced unduly by motion, even after motion correction. We took an approach similar to that described by Power et al. (2012), who removed images that were determined to be outliers on the basis of the six motion parameter estimates (MPEs) recorded for each subject *and* were displaced based on assessing voxel intensity changes in each brain volume, across each time course. We tested for outliers by identifying and removing time points that were outliers in both the six rigid-body MPEs, and in the fMRI signal using a multivariate approach. For an fMRI data matrix ***X***_ fmri_ (with dimensions *N*_voxels_ × *N*_time_) and a matrix of MPE time courses ***X***_mpe_ (6 × *N*_time_), we carried out the following adaptive and robust procedure on the pre-processed time courses for each participant:

1. We decomposed ***X***_fmri_ and ***X***_mpe_ using Principal Component Analysis, and represented the data in PC space coordinates, as ***Q***_fmri_ (with dimensions *N*_time_ × *N*_time_) and ***Q***_mpe_ (6 × *N*_time_). The PCA provides an orthonormal basis that maximizes the explained variance in the data, and greatly reduces the dimensionality of fMRI data.

2. For each PC-space data point ***q***_t_ (1≤*t*≤*N*_time_), we computed the median PC-space coordinate vector ***q***_med(_**_t__)_ in a 15-TR time window centered at *t*. We then obtained the squared Euclidean distance ${d=\left\|q_{t}-q_{med(t)}\right\|}^{2}$. This measures the displacement of ***q***_t_ away from surrounding data points; a point ***q***_t_ with larger displacement *d*_t_ is more likely to be an outlier. This procedure is performed for all data points in *** Q***_fmri_ and ***Q***_mpe_, producing vectors of displacement values ***d***_fmri_ and ***d***_mpe_, corresponding to time points in the fMRI data.

3. For each ***d***, we fit a Gamma probability distribution to the data, by computing the maximum likelihood estimates of the distribution parameters. The Gamma model was used, as it forms the distribution over a set of random, strictly positive variables. We then identified time points that were outliers at *p* <0.05, for both ***d***_fmri_ and ***d***_mpe_ distributions. These were labeled as motion outliers in the data.

4. We removed any outlier fMRI volumes, and replaced them by interpolating voxel values from adjacent volumes, using cubic splines. This controls for potential spikes, while minimizing discontinuities in the fMRI time courses due to removal of outliers.

### DATA ANALYSIS

The resting state data were analyzed with PLS (McIntosh and Lobaugh, 2004; Krishnan et al., 2011), a multivariate analysis technique that can identify whole-brain patterns of activity related to a predefined region or pair of regions (seed-PLS). This method is similar to principal component analysis, in that it identifies a set of principal components or “latent variables” (LVs) that optimally capture the covariance between two sets of measurements. In seed-PLS, each LV represents the pattern of correlation, or functional connectivity, between activity in a predefined region(s) and all other voxels in the brain. These correlations are calculated across subjects for each group (and for each seed, when using multiple seeds, as we did here) and then compared across groups/seeds. Each brain voxel has a weight, known as a salience, which indicates how strongly that voxel contributes to the LV overall. The significance of each LV as a whole was determined using a permutation test, using 500 permutations. In addition, the reliability of each voxel’s contribution to a particular LV was tested by submitting all saliences to a bootstrap estimation of the standard errors (SEs, Efron, 1981), using 100 bootstraps. Peak voxels with a salience/SE ratio ≥5.0 (the bootstrap ratio, or BSR, *p* <0.001) were considered to make a robust contribution to the LV. Clusters containing at least 10 above-threshold contiguous voxels were extracted, with a local maximum defined as the voxel with a BSR higher than any other voxel in a 2 cm cube centered on that voxel (the minimum distance between peaks was 10 mm). Coordinates of these locations are reported in MNI space.

We performed two seed-PLS analyses on the resting state data from both younger and older adults. One analysis examined the connectivity of two regions from the Andrews-Hanna paper (2010): the left parahippocampal gyrus (*X*: -28, *Y*: -40, *Z*: -12) and the dmPFC (*X*: -4, *Y*: 48, *Z*: 24). The other analysis examined the connectivity of two regions from the Leech paper (2011): the vPCC (*X*: 2, *Y*: -58, *Z*: 28) and the dPCC (*X*: 2, *Y*: -34, *Z*: 40). Despite our use of a relatively large voxel size (4 mm × 4 mm× 4 mm) the dorsal and ventral PCC seeds did not overlap and were readily distinguishable at a Euclidean distance of 2.68 cm. In preparation for these analyses, we first averaged each consecutive 5 volumes from the resting run, to produce 29 “blocks” of 10s each (excluding the first 5 TRs to allow for signal normalization). This averaging process effectively produced a low-pass filter of 0.1Hz and reduced temporal noise (Grigg and Grady, 2010a,b). Then for each time point, we extracted the mean signal from each seed voxel and then correlated the signal from both seeds (i.e., the MTL and dmPFC for the first analysis and the vPCC and dPCC for the second analysis) to all other voxels in the brain, across participants. To obtain summary measures of each participant’s expression of each LV pattern, we calculated “brain scores” by multiplying each voxel’s salience by the BOLD signal in the voxel, and summing over all brain voxels for each participant. This resulted in a brain score for each participant in each “block,” for each LV. To provide an assessment of functional connectivity, brain scores were correlated with each seed’s activity in each “block” and the bootstrap was used to calculate 95% confidence intervals around these correlations. If there is a common network to which both seeds are strongly connected, this common network should be identified in the first LV, as the first LV accounts for the most covariance in the data (with subsequent LVs accounting for progressively less covariance). The unique patterns of functional connectivity that differentiate the two seeds should be identified by the second LV.

We report the first two LVs from each analysis. For each LV we obtained a pattern of brain activity characterizing the regions with functional connectivity to the seeds and four sets of 29 correlations (one correlation per block): one set for each seed in the young group and one for each seed in the older group. Within-group differences in these correlation distributions were assessed using non-parametric Wilcoxon signed-rank tests, and between-group differences were assessed with Mann–Whitney *U* tests. Bonferroni corrections were applied based on the number of contrasts (two within-group or two between-group) for each LV.

## RESULTS

### FUNCTIONAL CONNECTIVITY OF THE MTL AND dmPFC

**FIGURE 1:**
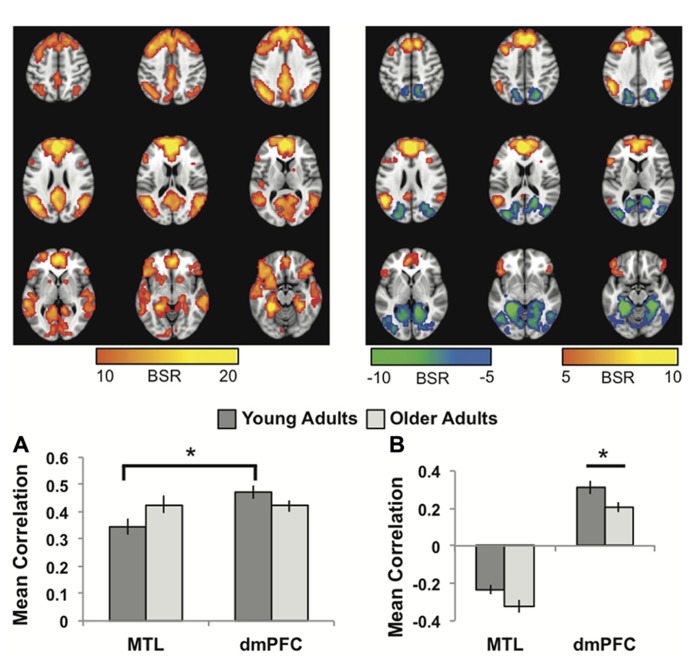
**(A)** The common network of regions positively correlated with MTL and dmPFC. **(B)** The regions uniquely correlated with the dmPFC (warm colors and positive correlations in the graph), or the MTL (cool colors and negative correlations in the graph). In both panels the graphs show the mean correlations (averaged across all 29 “blocks” in the resting run) between activity in each seed and the corresponding pattern of regions; i.e., the mean correlation between seed activity and the brain scores for young and older adults. In **(B)**, warm-colored regions (i.e., regions with positive weights on the LV) are positively correlated with the dmPFC and cool-colored regions (negative weights) are positively correlated with the MTL. Error bars represent standard errors of the mean for the correlations. The color bars refer to the range of BSR values seen in the brain images (a BSR threshold of 10 was used). Asterisks indicate significant differences in functional connectivity (*p* <0.05, corrected). In this figure and subsequent ones, the images shown are from 48 mm above the AC-PC line to 16 mm below this line, in 8 mm increments.

The first LV revealed a group of regions positively correlated with both MTL and dmPFC, in both age groups (*p* <0.001, 43.6% of the covariance). In addition to the seeds, this network included all of the regions typically included in the DMN, such as vmPFC, bilateral angular gyrus, anterior temporal lobes, and superior frontal gyri (**Figure 1A**; **Table 1**). Other regions correlating with the seeds included bilateral IFG and striatum. All of the correlations were strongly positive for both seeds and both age groups (**Figure 1A**), and there were no age differences, although there was a trend for the MTL to be more strongly connected with the common network in older adults (*p * = 0.03, uncorrected). The young adults showed weaker connectivity of the MTL with the common network, compared to that seen for the dmPFC (*p* <0.01, corrected), whereas the correlations were equivalent in the older adults.

**Table 1:** Common brain networks.

	MTL/dmPFC				vPCC/dPCC			
Region	*X* (mm)	*Y* (mm)	*Z* (mm)	BSR	*X* (mm)	*Y* (mm)	*Z* (mm)	BSR
Ventromedial PFC	4	48	-8	20.0	-4	40	-8	21.7
dmPFC	-*4*	*48*	*24*		0	52	24	19.2
R superior frontal gyrus	20	36	44	18.0	28	40	28	23.2
L superior frontal gyrus	-20	44	36	20.4	-24	36	28	15.7
R inferior frontal gyrus	40	28	-20	16.7	44	24	-20	13.1
L inferior frontal gyrus	-36	28	-16	15.8	-44	20	-20	13.2
R MTL	28	-40	-12	17.5	20	-28	-8	12.4
L MTL	-*28*	-*40*	-*12*		-16	-36	-4	14.4
R middle temporal gyrus	56	-36	-8	15.6	60	-36	0	18.3
L middle temporal gyrus	-60	-32	0	17.7	-60	-48	8	18.4
R angular gyrus	52	-64	20	18.5	44	-64	28	20.9
L angular gyrus	-44	-64	20	21.1	-40	-68	24	16.7
Cerebellum	28	-72	-36	12.0	-4	-60	-28	13.3
R striatum	12	8	-4	12.4	12	8	0	14.0
vPCC	-8	-48	24	18.1	*4*	-*56*	*28*
dPCC	0	-32	40	17.9	*4*	-*32*	*40*	

**FIGURE 2:**
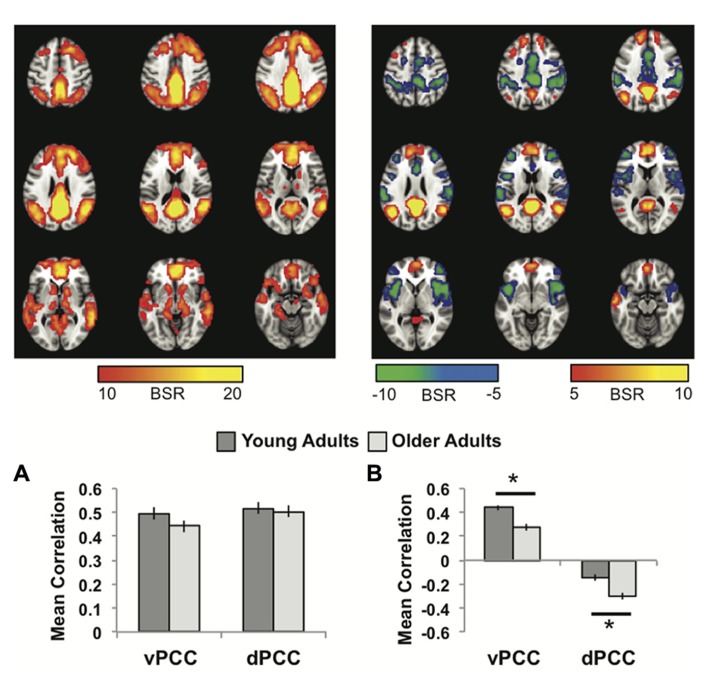
**(A)** The common network of regions correlated with vPCC and dPCC. **(B)** The regions uniquely correlated with the vPCC (warm colors and positive correlations in the graph) or the dPCC (cool colors and negative correlations in the graph). In both panels the graphs show the mean correlations (averaged across all 29 “blocks” in the resting run) between activity in each seed and the corresponding pattern of regions; i.e., the mean correlation between seed activity and the brain scores for young and older adults. Error bars represent standard errors of the mean for the correlations. The color bars refer to the range of BSR values seen in the brain images (a BSR threshold of 5 was used). Asterisks indicate significant age group differences in functional connectivity (*p* <0.05, corrected).

*Clusters shown in **Figures 1A** and **2A**. BSR = bootstrap ratio. Seed regions are indicated with italics.*

The second LV (*p* <0.001, 6.1% of the covariance) identified distinct regions that robustly correlated either with the dmPFC (warm colors and positive correlations in **Figure 1B**), or with the MTL (cool colors and negative correlations in **Figure 1B**). The dmPFC was correlated with the PCC and inferior parietal lobes, with maxima in the supramarginal gyri, extending into the angular gyri. Non-DMN frontal areas also were part of the dmPFC subsystem. This set of regions is similar to the pattern for the dmPFC noted previously (Andrews-Hanna et al., 2010). The regions showing functional connectivity to the left MTL were similar to those identified by Andrews-Hanna et al. (2010), and include retrosplenial cortex, right MTL, and posterior/medial parietal regions (**Table 2**). Areas of occipital cortex also correlated with MTL. Thus both the MTL and dmPFC subsystems included regions outside the DMN, consisting of occipitoparietal areas for the MTL and frontal regions for the dmPFC.

**Table 2:** Brain regions involved in each subsystem.

Region	*X* (mm)	*Y* (mm)	*Z* (mm)	BSR
**dmPFC SUBSYSTEM**
L middle frontal gyrus	-36	20	36	9.4
R inferior frontal gyrus	36	20	28	6.0
L inferior frontal gyrus	-48	44	-12	8.2
L posterior cingulate gyrus	-4	-44	24	5.9
R supramarginal gyrus	56	-52	32	7.1
L supramarginal gyrus	-52	-52	32	11.3
**MTL SUBSYSTEM**
R MTL	28	-36	-16	-13.6
R posterior intraparietal sulcus	24	-64	36	-8.1
L posterior intraparietal sulcus	-20	-64	32	-8.1
R middle occipital gyrus	44	-76	16	-8.7
L middle occipital gyrus	-36	-84	16	-9.0
R retrosplenium	20	-52	16	-10.5
**vPCC SUBSYSTEM**
R superior frontal gyrus	20	40	40	7.5
Ventromedial PFC	-4	52	12	9.2
L angular gyrus	-44	-64	24	13.1
R angular gyrus	56	-60	20	12.6
L middle temporal gyrus	-60	-4	-20	10.0
R middle temporal gyrus	56	-4	-24	6.9
L middle frontal gyrus	-36	16	48	5.9
R cerebellum	28	-76	-36	7.7
**dPCC SUBSYSTEM**
R anterior insula/frontal operculum	40	20	0	-9.9
L anterior insula/frontal operculum	-40	12	-4	-9.7
R middle frontal gyrus	36	44	24	-9.3
L inferior frontal gyrus	-44	44	4	-8.3
R dorsal anterior cingulate gyrus	4	8	40	-9.7
R supramarginal gyrus	60	-36	40	-11.1
L supramarginal gyrus	-60	-36	28	-10.2

*Clusters shown in **Figures 1B** and **2B**. BSR = bootstrap ratio.*

In older adults these two distinct subsystems of functional connectivity were maintained. However, the dmPFC was more weakly correlated with its network in older adults, compared to younger adults (*p* = 0.03, corrected). The MTL showed a trend for a larger correlation with its network in older adults, but the age difference was not significant (*p* = 0.08 uncorrected).

### FUNCTIONAL CONNECTIVITY OF THE vPCC AND dPCC

The first LV of this analysis (*p* <0.001, 50.3% of the covariance) identified regions commonly correlated with both seeds, which included all other regions of the DMN, as well as caudate and thalamus bilaterally (**Figure 2A**; **Table 1**). As with the MTL/dmPFC analysis, there were no age differences in the correlations of the vPCC and dPCC with this network (**Figure 2A**), and there also were no significant differences in the strength of the correlations between the two seeds within either age group.

The second LV (*p* <0.001, 5.6% of the covariance) showed distinct regions with functional connectivity to the vPCC (warm colors and positive correlations in **Figure 2B**) and dPCC (cool colors and negative correlations in **Figure 2B**). The unique network for the vPCC included only other DMN regions, such as superior frontal gyri, angular gyri, and vmPFC. The unique network for the dPCC included bilateral supramarginal gyri, middle frontal gyri, anterior insula/frontal opercular regions, and dorsal anterior cingulate (**Table 2**). These regions are typically included in two task-related networks, i.e., the salience and frontoparietal control networks (Seeley et al., 2007; Vincent et al., 2008). These different patterns are in line with what was suggested, but not shown, by Leech et al. (2011). Overall, this LV supports the idea that the vPCC is more closely connected to the default network and the dPCC interacts with both the default and task positive networks.

Older adults showed a similar pattern of connectivity (**Figure 2B**), but age differences were seen in the strength of the correlations. Older adults had weaker functional connectivity in the vPCC subsystem (*p* <0.001, corrected), but stronger correlations within the dPCC subsystem (*p* = 0.004, corrected).

### SIMILARITIES AND DIFFERENCES BETWEEN THE COMMON NETWORKS AND SUBSYSTEMS

**FIGURE 3:**
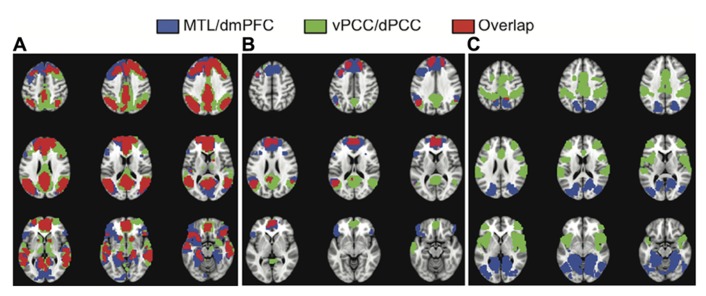
**The overlap between the common networks (LV1) (A), the dmPFC and vPCC subsystems (from LV2) (B), and the MTL and dPCC subsystems (from LV2) (C).** There is considerable overlap between the common networks identified in the two analyses, including all major nodes of the DMN **(A)**. There also is overlap in DMN regions between the dmPFC and vPCC subsystems, including superior frontal and angular gyri, in addition to the two seeds **(B)**. There is essentially no overlap between the MTL and dPCC subsystems **(C)**. A BSR threshold of 10 was used in **(A)** and a threshold of 5 was used in **(B,C)**.

To assess the similarity of the functional connectivity patterns we calculated the correlation between the unthresholded brain images for LV1 obtained from each analysis, and the correlation for the LV2 images. As would be expected if all four seed regions are part of the DMN, and the first LVs represent the full DMN, there was a strong correlation between the images identified in first LVs (*r* = 0.77, bootstrapped confidence interval = 0.78, 0.76). In addition, there was a large degree of spatial overlap between the common network seen for the vPCC/dPCC, and the one identified for the MTL/dmPFC (red regions in **Figure 3A**). These regions of overlap included the four seed regions, as well as vmPFC, and bilateral angular gyrus, superior frontal gyrus, anterior temporal cortex, and caudate. Although this overlap indicated marked similarity between these common networks, some differences also were noted. The common network for vPCC and dPCC included the thalamus and areas of right prefrontal cortex not seen for the MTL/dmPFC (green regions in **Figure 3A**). In addition, the MTL/dmPFC showed more extensive correlations with ventral frontal cortex and right MTL than did the vPCC/dPCC.

**Figures 1** and **2** suggest that the dmPFC and vPCC subsystems include a number of regions in common, although the MTL and dPCC subsystems seem to be relatively distinct. Accordingly, the correlation between the unthresholded images corresponding to these second LVs was less than seen for the first LVs (*r* = 0.29, bootstrapped confidence interval = 0.31, 0.28). This correlation was likely due to the similarity between the dmPFC and vPCC subsystems (**Figure 3B**). Overlap between the dmPFC and vPCC subsystems included the two seed regions, as well as bilateral parietal cortex, superior frontal gyri, and left dorsolateral PFC. Despite this overlap, the parietal regions maximally involved in the dmPFC subsystem (supramarginal gyri) were anterior to those involved in the vPCC subsystem (angular gyri, see **Table 2**). In contrast, there was no overlap between the MTL and dPCC subsystems (**Figure 3C**), with the exception of a small region in right medial parietal cortex. This suggests that these two latter subsystems are truly unique, whereas the dmPFC and vPCC subsystems share some regions, in addition to showing some unique features.

## DISCUSSION

In the present study, we used a multivariate seed analysis that allowed us to directly compare the full functional connectivity patterns of multiple seeds, as well as age groups, and showed that nodes within the default network do indeed have both common and distinct connectivity patterns. Across both analyses, the common network was the strongest pattern in the data, accounting for the most covariance, and resembled the canonical default network. The two distinct functional connectivity patterns identified by the MTL and dmPFC analysis look similar to those identified by Andrews-Hanna et al. (2010). Both subsystems include other DMN regions, as well as non-DMN areas. The two distinct functional connectivity patterns identified by the vPCC and dPCC analysis were similar to those identified by Leech et al. (2011), and can be described as a stronger relation between the vPCC and the DMN on the one hand, and between the dPCC and task relevant regions on the other. Thus, our findings provide further support for the conclusions of these two previous papers, and provide direct evidence to support suggestions made in those papers. In terms of comparing young and older adults, we found that older adults show the same pattern of functional connectivity for both the common network and subsystems, but the strength of connectivity within these subsystems differs with age. Weaker functional connectivity in two subsystems was found (dmPFC and vPCC), but stronger correlations were seen in the dPCC subsystem. Below we discuss the contribution of these results to understanding these DMN subsystems, and the implications of the age differences observed in them.

### THE DMN SUBSYSTEMS

Multi-seed PLS allowed us to directly compare, in a single analysis step, the whole-brain pattern of connectivity of multiple seeds to uncover both their common and unique patterns of connectivity. This direct comparison of overall functional connectivity between specific seeds cannot be accomplished by either conventional univariate seed analyses, which show the within-subject connectivity pattern of one seed at a time, or analyses that are purely data driven (e.g., ICA) and do not provide a means to contrast the connectivity patterns of particular regions. Thus, in addition to replicating previous results, our findings extend the earlier work in several ways. First, the amount of covariance accounted for by the common functional connectivity patterns (40–50%) was considerably larger than that of the unique functional connectivity patterns (~6%). This indicates that these regions are much more strongly connected with the full DMN at rest than they are with their respective subsystems, although this need not be the case for task situations (e.g., Leech et al., 2011). However, we also found that the MTL was less robustly functionally coupled with the default network than the dmPFC in younger adults, which may explain why the MTL is only sometimes reported as part of the DMN in the literature (e.g., Buckner et al., 2008; Preminger et al., 2011). Second, the subsystems are more spatially extensive than previously described (Andrews-Hanna et al., 2010; Leech et al., 2011), reflecting the whole-brain approach that we took, as well as the greater sensitivity of this multivariate statistical approach relative to univariate models (e.g., Fletcher et al., 1996; Lukic et al., 2002). This highlights the point that determining the regions participating in any brain “network” will depend to some extent on the method one uses to identify the regions (Grigg and Grady, 2010a; Yourganov et al., 2011). Finally, the MTL and dPCC subsystems appear to be largely non-overlapping, whereas those of the vPCC and dmPFC do overlap to some extent, mainly because they include the same DMN regions. This suggests the interesting possibility that the vPCC subsystem is not really a “subsystem” at all, but rather the set of DMN regions with the strongest functional interconnections, whereas the other three areas do participate in other networks consisting of regions within and outside of the DMN.

Although our results do not speak to the function of these different subsystems, previous work does suggest a functional dissociation between them. Andrews-Hanna et al. (2010) showed that the dmPFC subsystem is preferentially active when making a decision about oneself in the present, while the MTL subsystem is preferentially active when making a decision about oneself in the future, presumably because future projection also makes use of the episodic memory system (Schacter et al., 2007). They further showed that the vmPFC-PCC midline core is active during either form of self-referential thought. A recent meta-analysis also suggests a distinction between a core midline subsystem (including the vmPFC and PCC), which primarily mediates self-referential processing, and a parieto-temporal subsystem (including the inferior parietal lobule, MTL, and lateral temporal cortex) which is associated with memory retrieval processes (Kim, 2012). While the subsystems outlined in these two earlier papers are not a perfect match, both studies emphasize the point that separate subcomponents of the default network, which are typically correlated at rest and deactivated together during most tasks, can be differentially activated depending on the nature of the task, and likely support different aspects of self-referential processing. Our results would be in line with this idea, and further suggest that the MTL subsystem would involve more visual processing and/or imagery than the dmPFC system, given the extensive occipital regions co-active with the MTL, perhaps reflecting the role of the MTL in scene construction (Hassabis and Maguire, 2007; Schacter and Addis, 2007).

The functional distinction between the vPCC and dPCC subsystems seems more general, in that the vPCC subsystem included primarily a subset of DMN regions, suggesting that it is more integrated with the default network as a whole, while the dPCC subsystem involved areas thought to be nodes of several task positive networks. Similarly, Leech et al. (2011) showed that the integration of these two regions with the DMN depends on the level of task demand, such that during an easy task, the vPCC showed strong integration with the default network and the dPCC showed integration with both the default network and cognitive control network. The authors suggest that the dPCC may modulate the dynamic interaction between the default and attention networks. The present results are consistent with this idea, and also suggest that the dPCC is correlated to an even wider group of task positive regions, including areas involved in salience processing (Seeley et al., 2007). Of course, our results reflect intrinsic functional connectivity during rest and the dPCC may be differentially connected with these task-positive areas during task performance.

### AGE DIFFERENCES IN DMN SUBSYSTEMS

Our findings with older adults suggest that the common networks are not substantially affected by age, suggesting that these extensive patterns of functional connectivity involving the entire DMN, and a few other areas, are relatively stable until the 70s or early 80s. This is not to say, however, that there are no age differences in the DMN, as we found that the individual subsystems are vulnerable to aging. Younger adults showed stronger connectivity within the dmPFC and vPCC subsystems. Insofar as these subsystems bear a resemblance to the classic default network, and both include each other as well as other DMN regions, this finding replicates previous demonstrations of an age difference in functional connectivity involving nodes of the DMN (e.g., Andrews-Hanna et al., 2007; Damoiseaux et al., 2008; Esposito et al., 2008; Grady et al., 2012).

More intriguing, older adults’ correlation values for the MTL showed a trend for stronger connectivity, and for the dPCC subsystem older adults had significantly stronger functional connectivity than did younger adults. Both of these subsystems consisted mostly of regions outside the DMN, such as posterior visual cortex for the MTL and frontoparietal control regions for the dPCC. This result for the MTL, although not robust, is consistent with a recent study that assessed functional connectivity with magnetoencephalography and showed that aging is associated with an *increase* in the inflow of information from sensory regions to the MTL (Schlee et al., 2012). Our result is also consistent with evidence that healthy aging affects MTL structure and function much less than is seen with AD (Buckner, 2004). At a behavioral level, this increased influx of information to the MTL may reflect older adults’ lessened ability to suppress distracting information (e.g., Stevens et al., 2008), which ultimately leads to their greater encoding of irrelevant associations (Campbell et al., 2010,b). An interesting question for future research is how age-related differences in memory function relate to the integrity of this subsystem.

In addition, greater functional connectivity between the dPCC and task-relevant regions, such as prefrontal cortex, in older adults replicates our earlier finding of stronger correlations within such regions during task performance in older adults (Grady et al., 2010). Stronger functional connectivity within prefrontal control regions also is consistent with the many reports of greater activation in these areas in older compared to younger adults (e.g., Grady et al., 1994; Cabeza et al., 1997; for a review, see Cabeza and Dennis, 2012), which often is associated with better task performance in the older individuals and considered to be compensatory (but see de Chastelaine et al., 2011). Our results suggest that altered functional connectivity involving control regions, as well as increased activation, may both be compensatory mechanisms, although this would need to be tested under task conditions. Taken together with consistent findings of weaker functional connectivity within the DMN, the current work supports the idea that the correlations among DMN nodes appears to be quite sensitive to the effects of age, especially when focusing on the connectivity of the vPCC (as many studies have done) and dmPFC, whereas other, more task-relevant, networks may only show age reductions in functional connectivity under experimental conditions that place heavy demands on them (Campbell et al., 2012a). Interestingly, it may be that reduced functional connectivity in DMN subsystems has as much, if not more impact on cognition in older adults than maintained connectivity in task-related networks. Several studies have reported that weaker functional connectivity or reduced modulation of activity in the PCC is associated with poorer cognitive performance in older adults (Andrews-Hanna et al., 2007; Miller et al., 2008; Park et al., 2010) and a recent study in younger adults found that resting functional connectivity of the dmPFC with the rest of the DMN was specifically related to deactivation of the DMN during an attention demanding task and to performance on the task (Dang et al., 2013). Our finding of reduced functional connectivity involving the dmPFC and vPCC in older adults suggests that such links with both subsystems could be disrupted in older age, leading to a lessened ability to suppress this system and greater interference during task performance (Grady et al., 2006). Additionally, these age differences involving resting functional connectivity in the dmPFC subsystem mediating self-related processing provide further evidence that such processing is altered with age (e.g., older adults often judge themselves more positively relative to younger adults; Grady, 2012). Indeed these two phenomena, reduced dmPFC functional connectivity and more positive outlook, may be related (Saverino et al., unpublished), although determining whether the former is a cause of the latter will require longitudinal research or lifespan studies.

Although all of our older participants scored in the normal range on the MMSE, we cannot rule out the possibility that some of our older cohort may be in the preclinical stages of AD. This may be relevant in that even cognitively normal older adults with evidence of amyloid deposition (i.e., are PIB+) show reduced functional connectivity within the DMN relative to those who are PIB- (Sheline et al., 2010). However, that study did not include a younger control group and thus, cannot speak to how “normal” or non-clinical aging affects the DMN. An earlier study by Andrews-Hanna et al. (2007) did address this issue and suggested that even normal aging is associated with reduced connectivity within the DMN, as the distributions of PIB- and PIB+ older adults overlapped in that study and were both lower than younger adults. In addition, our findings of maintained MTL functional connectivity and increased dPCC connectivity both suggest that our results are not heavily influenced by incipient AD pathology. The vast majority of studies looking at DMN functional connectivity in the presence of amyloid, or in AD or mild cognitive impairment (MCI), have found reduced connectivity, particularly involving the MTL (e.g., Greicius et al., 2004; Hedden et al., 2009; Sperling et al., 2010; Petrella et al., 2011). Thus, the pattern of age effects across subsystems that we observed does not appear to be easily explained by inclusion of individuals with preclinical disease. Nevertheless, our results may be affected by the inclusion of some individuals with preclinical AD, as is true of all aging research.

Finally, it has recently been suggested that functional connectivity estimates are particularly vulnerable to motion artifact and that group differences in connectivity could be influenced by confounding differences in head motion between groups (Power et al., 2012; Van Dijk et al., 2012). We show here that age differences in functional connectivity, both decreases and increases, can be observed after rigorously removing images influenced by head motion. It also is likely that averaging across time points, and assessing correlations across participants, as we did here, will lessen the effects of any artifact that affects single, or only a few contiguous volumes in a given person’s time series. Nevertheless, it seems clear that careful quality control of image data and some form of “scrubbing” should be routine components of fMRI analysis, especially for functional connectivity analyses that depend on voxel-wise correlations of time series within-subject.

In conclusion, we were able to directly contrast the connectivity patterns of multiple seeds by using a multivariate analysis technique. In line with recent work, we found that individual nodes within the default network are functionally connected to both a common network and individual subsystems, consistent with the idea that these subsystems have different functional roles. We further showed that older adults’ functional connectivity patterns for each of these seeds resembled those of younger adults, with two showing reduced connectivity in older adults (dmPFC and vPCC) and the other two showing maintained (MTL) or increased connectivity with age (dPCC). These findings suggest that future studies of aging and default network connectivity at rest and during tasks should examine individual subsystems to gain a fuller picture of age-related change.

## Conflict of Interest Statement

The authors declare that the research was conducted in the absence of any commercial or financial relationships that could be construed as a potential conflict of interest.

## Footnotes

1 The data were combined after determining that the patterns of functional connectivity were very similar across the three experiments.
